# Characteristics and survival of adults with differentiated thyroid cancer in a Peruvian hospital

**DOI:** 10.17843/rpmesp.2024.413.13378

**Published:** 2024-08-28

**Authors:** Luz Morales-Concha, Iván Huamani-Linares, Katy Saihua-Palomino, Edward Luque Florez, Alexi Chávez Echevarría, Ramiro Jorge Tupayachi Palomino, Carlos Antonio Zea Nuñez, Christian R. Mejia, Noé Atamari-Anahui

**Affiliations:** 1 Faculty of Human Medicine, Universidad Nacional de San Antonio Abad del Cusco, Cusco, Peru. Universidad Nacional San Antonio Abad del Cusco Faculty of Human Medicine Universidad Nacional de San Antonio Abad del Cusco Cusco Peru; 2 ASOCIEMH-CUSCO, Faculty of Human Medicine, Universidad Nacional de San Antonio Abad del Cusco, Cusco, Peru. Universidad Nacional San Antonio Abad del Cusco ASOCIEMH-CU Faculty of Human Medicine Universidad Nacional de San Antonio Abad del Cusco Cusco Peru; 3 Department of General Surgery, Hospital Antonio Lorena, Cusco, Peru. Department of General Surgery Hospital Antonio Lorena Cusco Peru; 4 Adolfo Guevara Velasco National Hospital - EsSalud, Cusco, Peru. Adolfo Guevara Velasco National Hospital - EsSalud Cusco Peru; 5 Universidad Continental, Huancayo, Peru. Universidad Continental Huancayo Peru; 6 Medical Association of Research and Health Services, Lima, Peru. Medical Association of Research and Health Services Lima Peru; 7 Research Unit for the Generation and Synthesis of Health Evidence, Vice Rectorate for Research, San Ignacio de Loyola University, Lima, Peru. San Ignacio de Loyola University Research Unit for the Generation and Synthesis of Health Evidence Vice Rectorate for Research San Ignacio de Loyola University Lima Peru

**Keywords:** Thyroid Neoplasms, Survival Rate, Mortality, Peru

## Abstract

This study aimed at studying the clinical and anatomopathological characteristics, treatment and survival of patients with differentiated thyroid cancer. A retrospective cohort study was conducted with data from 150 patients from a Peruvian hospital between the years 2010 to 2020. Characteristics and survival (Kaplan-Meier method) were described. The mean age was 48.3 years, 130 participants (86.7%) were women and the most frequent histologic type was papillary 94.6%. Of the participants, 74.2% had TNM stage I, 70.7% had total thyroidectomy and 68.7% received radioactive iodine. Overall survival at 5 years was 89.3%, being lower in those with TNM stage IV and higher in those who used radioactive iodine. In conclusion, in a hospital in Cusco, differentiated thyroid cancer was more frequent in women and survival was lower compared to reports from other countries.

## INTRODUCTION

Thyroid cancer is the most frequent neoplasm of the endocrine system ^1^. According to the histological type it can be categorized into differentiated, medullary and anaplastic types ^1^. Differentiated thyroid cancer accounts for 90% of all cases and includes papillary and follicular types ^2^.

According to the GLOBOCAN study, which compiled thyroid cancer registries from 185 countries, by the year 2020, the overall incidence was higher in women than in men (10.1 cases vs. 3.1 cases per 100,000 population-years) ^3^; as was mortality (0.5 deaths per 100,000 population in women and 0.3 deaths per 100,000 population in men) ^3^. In Peru, an incidence of 6.3 cases per 100,000 population-years was estimated by 2022, ranking tenth among all cancers in general and a mortality of 0.9 deaths per 100,000 population ^4^.

This is due to the increase in the incidence of thyroid cancer worldwide, possibly because of the detection of small and low-risk tumors as a consequence of increased surveillance, but also due to the presence of some risk factors, such as exposure to environmental or therapeutic ionizing radiation, family history and obesity ^2^. Treatment in adults is early and timely, resulting in greater medium and long-term survival. Survival of differentiated thyroid cancer is greater than 95% at five years ^5^, and up to 15 years ^6^; however, there may be persistence or recurrence of the cancer during follow-up, which generates intensive surveillance and other treatment options ^7^.

There are studies of this type of cancer in Peru that are limited to the description of this neoplasm at diagnosis ^8^^-^^10^, leaving a gap in the evolution and survival of patients, which could have relevance particularly for those with recurrence of the disease, advanced stage or metastasis. Due to the increasing incidence of this neoplasm, we decided to conduct this study with the aim of describing the characteristics of patients with differentiated thyroid cancer and survival in a hospital in Cusco.

**KEY MESSAGES**

Motivation for the study. There are few clinical and survival studies in Peru on thyroid cancer.

Main findings. Between the years 2010 to 2020, differentiated thyroid cancer was more frequent in women with early-stage disease, but survival was lower at five years compared to reports from other countries.

Implications. Thyroid cancer has increased in recent decades worldwide. It is important to have specialized and decentralized centers for the initial management and follow-up of these patients to avoid long-term complications or fatal outcomes and to have updated epidemiological information.

## THE STUDY

### Design and population

We conducted a retrospective cohort study with data from the medical records of patients with thyroid cancer diagnosed for the first time at the Adolfo Guevara Velasco National Hospital of Cusco (belonging to the Peruvian social security); between January 1, 2010 to December 31, 2016, with treatment and follow-up until December 31, 2020.

All medical records of patients older than 18 years with histological confirmation of differentiated thyroid cancer (papillary and follicular) in the pathology department of the hospital were included. We excluded 20 patients out of 170 patients in the study period: 17 patients initially from other institutions and three with different histological type (2 medullary and 1 anaplastic). Sampling was consecutive for all eligible patients, with a final sample of 150 medical records.

### Study variables

We studied sociodemographic variables such as sex (female and male), age at diagnosis (20-54 and ≥ 55 years), clinical manifestations (neck pain, dysphagia, dysphonia, dyspnea and other symptoms not related to thyroid cancer), characteristics of the physical examination of the thyroid (no alterations, thyroid nodule and multinodular goiter), initial thyroid function (euthyroid, hypothyroid and hyperthyroid) through the measurement of thyroid stimulating hormone, free thyroxine and triiodothyronine.

We also evaluated anatomopathological characteristics such as tumor size, histologic type (papillary or follicular), extrathyroid extension (yes or no), regional lymph node metastasis (yes or no), distant metastasis (yes or no) and TNM stage (tumor, lymph nodes and metastasis) eighth edition (I, II, IV) ^11^, treatment characteristics such as type of surgery (total thyroidectomy or lobectomy), cervical lymphadenectomy (yes or no), use of radioactive iodine (yes and no), post-surgical characteristics (hypothyroidism, hypoparathyroidism and laryngeal nerve injury), and post-surgical characteristics (hypothyroidism, hypoparathyroidism and laryngeal nerve injury), cervical lymphadenectomy (yes or no), use of radioactive iodine (yes or no), post-surgical characteristics (hypothyroidism, hypoparathyroidism and recurrent laryngeal nerve injury), follow-up characteristics (persistence, recurrence or excellent response to treatment), survival, which was defined as the duration (days) from the date of diagnosis to cancer-associated death and final status (deceased or alive).

### Procedures and statistical analysis

Once the included 150 medical records had been identified, the information was reviewed and collected from the hospital archive area using a data collection form after approval and authorization of the project by the hospital’s institutional research committee. The information was then deposited in a Microsoft Excel ® for Windows 10 database. Once the database was completed, it was reviewed by two researchers and then the information was processed in the Stata v.17 program (StataCorp LP, College Station, TX, USA).

Categorical variables were described by frequencies (absolute and relative), and numerical variables by measures of central tendency (mean or median) and dispersion (range and standard deviation); this according to the previous evaluation of the normality of the data by means of the Shapiro-Wilk statistical test. Comparisons were made according to sex and between the two histological types (papillary and follicular) by means of the Chi-square or Fisher’s exact test for categorical variables and Student’s t-test or Mann-Whitney U test for numerical variables; those with a p-value <0.05 were considered significant. Survival time was censored for participants alive at the end of the study (December 31, 2020) and those who died of other causes (2 participants). Survival was estimated by the Kaplan-Meier method and the log-rank test was used to evaluate the differences between groups, considering a p-value <0.05.

### Ethical aspects

The project was approved by the ethics committee of the Hospital Nacional Adolfo Guevara Velasco de Cusco (resolution N°74 -GRACU-ESSALUD-2020). An anonymous list was used before conducting the analysis to avoid identifying the participants.

## FINDINGS

Of the 150 patients included in the study, 130 (86.7%) were female and the female/male ratio was 6.5. The mean age at diagnosis was 48.3 (SD: 12.7 years). Regarding clinical characteristics, 39 (26%) had neck pain, 23 (15.3%) dysphagia, 19 (12.7%) dysphonia, 12 (8%) dyspnea and 83 (55.3%) other symptoms not related to thyroid cancer. On physical examination, 46 (30.7%) had unaltered thyroid, 59 (39.3%) thyroid nodule and 45 (30.0%) multinodular goiter. Regarding thyroid function, 92 (61.3%) were euthyroid, 53 (35.3%) had hypothyroidism and five (3.3%) hyperthyroidism. The median primary tumor size was 2.5 cm (interquartile range 1.5 to 3.7), 51 (34%) had extrathyroidal extension, 86 (57.3%) had regional lymph node metastases, 23/128 (18%) had distant metastases and 95 (74.2%) had TNM stage I (eighth edition).

Age at diagnosis was higher in men than in women (56.5 years vs. 47 years, p=0.002). Papillary was the most frequent histologic type in 142 (94.7%) patients and follicular in 8 (5.3%) patients. No significant differences were found between tumor size, extrathyroidal extension, regional lymph node metastasis, distant metastasis and TNM stage (Table 1).

**Table 1:** Clinical characteristics of patients with differentiated thyroid cancer according to histologic type and sex.

Characteristics		Papillary	Follicular	p-value ^c^	Male	Female	p-value ^c^
		n (%)	n (%)		n (%)	n (%)	
Sex				0,598			
	Female	122 (85.9)	8 (100.0)		--	--	
	Male	20 (14.1)	0 (0.0)		--	--	
Age at diagnosis ^a^		48.4 (12.9)	47.1 (9.3)	0.792 ^d^	56.5 (11.9)	47 (12.4)	0.002 ^d^
	20-54	96 (67.6)	7 (87.5)	0.436	9 (45.0)	94 (72.3)	0.014
	≥ 55	46 (32.4)	1 (12.5)		11 (55.0)	36 (27.7)	
Tumor size ^b^		25 (15-35)	24 (19-41,5)	0.579 ^e^	25.5 (20-42.5)	25 (15-35)	0.218 ^e^
	≤ 4.0 cm	120 (84.5)	6 (75.0)	0.615	15 (75.0)	111 (85.4)	0.321
	> 4.0 cm	22 (15.5)	2 (25.0)		5 (25.0)	19 (14.6)	
Extra thyroid extension				0.717			0.265 ^f^
	No	93 (65.5)	6 (75.0)		11 (55.0)	88 (67.7)	
	Yes	49 (34.5)	2 (25.0)		9 (45.0)	42 (32.3)	
Metastasis to regional lymph nodes				1.000			0.456
	No	61 (43.0)	3 (37.5)		7 (35.0)	57 (43.9)	
	Yes	81 (57.0)	5 (62.5)		13 (65.0)	73 (56.1)	
Distant metastases (n=128)				0.154			0.471
	No	100 (83.3)	5 (62.5)		11 (73.3)	94 (83.2)	
	Yes	20 (16.7)	3 (37.5)		4 (26.7)	19 (16.8)	
TNM Stage (n=128)				0.260			0.056
	I	90 (75.0)	5 (62.5)		9 (60.0)	86 (76.1)	
	II	17 (14.2)	3 (37.5)		2 (13.3)	18 (15.9)	
	IVA	1 (0.8)	0 (0.0)		1 (6.7)	0 (0.0)	
	IVB	12 (10.0)	0 (0.0)		3 (20.0)	9 (8.0)	

a Mean (standard deviation), ^b^ Median (interquartile ranges), ^c^ Fisher’s exact test, ^d^ Student’s t-test, ^e^ Mann-Whitney U test, ^f^ Chi-square test.

Regarding treatment, 106 (70.7%) had total thyroidectomy, 63 (42%) had neck dissection with lymphadenectomy (31 had modified radical dissection, 22 selective dissection, 20 lateral and 2 posterolateral dissection, and 10 had central dissection). After surgery, 146 (97.3%) had hypothyroidism, 58 (38.7%) had hypoparathyroidism and 23 (15.3%) had recurrent laryngeal nerve injury. One hundred and three (68.7%) patients received radioactive iodine.

The median follow-up time was 4.6 years (ICER 3.5-6.6), it was 4.9 years (IQR: 3.9-6.8) for the group that completed follow-up (alive) and 1.9 years (IQR: 1-2) for those who presented the outcome (deceased).

At the end of follow-up, 15 deaths due to cancer were reported (13 papillary and 2 follicular). Of these, 11 (73.3%) were ≥ 55 years, all had regional lymph node metastases and 12 (85.7%) had distant metastases (Table 2). Of the 13 deceased patients with papillary cancer, all had regional lymph node metastases and 10 distant metastases, and the two deceased patients with follicular cancer also had these features. Of the 133 patients who did not have the outcome (alive), 44 (33.1%) had cancer persistence, 21 (15.8%) had recurrence and 68 (51.1%) had excellent response to treatment.

**Table 2:** Survival of patients with differentiated thyroid cancer.

Characteristics		Cases	Cancer deaths	Probability of survival	Log-rank
		n (%)	n (%)	S(t) 95%CI	
Sex					
	Female	130 (86.7)	11 (73.3)	90.9 (84.2-94.9)	0.121
	Male	20 (13.3)	4 (26.7)	79.0 (53.2-91.5)	
Age at diagnosis					
	20-54	103 (68.7)	4 (26.7)	95.8 (89.0-98.4)	<0.001
	≥ 55	47 (31.3)	11 (73.3)	75.0 (59.4-85.3)	
Histological type					
	Papillary	142 (94.7)	13 (86.7)	87 (76.6-93.0)	0.175
	Follicular	8 (5.3)	2 (13.3)	56.3 (14.7-84.2)	
Tumor size					
	≤ 4.0 cm	126 (84.0)	7 (46.7)	94.0 (87.8-97.1)	0.001
	> 4.0 cm	24 (16.0)	8 (53.3)	63.1 (39.3-79.7)	
Extrathyroid extension					
	No	99 (66.0)	4 (26.7)	95.4 (88.2-98.3)	<0.001
	Yes	51 (34.0)	11 (73.3)	77.4 (62.9-86.8)	
Metastasis to regional lymph nodes					
	No	64 (42.7)	0 (0.0)	100 (100.0)	<0.001
	Yes	86 (57.3)	15 (100.0)	81.6 (71.2-88.5)	
Distant metastasis (n=128)					
	No	105 (82.0)	2 (14.3)	98.0 (92.3-99.5)	<0.001
	Yes	23 (18.0)	12 (85.7)	45.8 (24.7-64.6)	
TNM stage (n=128)					
	I	95 (74.2)	1 (7.2)	99.0 (92.8-99.9)	<0.001
	II	20 (15.6)	3 (21.4)	81.7 (53.1-93.7)	
	IV	13 (10.2)	10 (71.4)	23.1 (5.6-47.5)	
Surgery					
	Total thyroidectomy	106 (70.7)	12 (80.0)	87.9 (79.6-92.9)	0.42
	Lobectomy	44 (29.3)	3 (20.0)	92.6 (78.7-97.5)	
Lymphadenectomy					
	No	87 (58.0)	4 (26.7)	95.1 (87.5-98.1)	0.01
	Yes	63 (42.0)	11 (73.3)	81.3 (68.7-89.2)	
Radioactive iodine					
	No	47 (31.3)	11 (73.3)	74.6 (58.6-85.1)	<0.001
	Yes	103 (68.7)	4 (26.7)	95.9 (89.4-98.4)	

TNM: tumor, node and metastasis.

**Figure 1:**
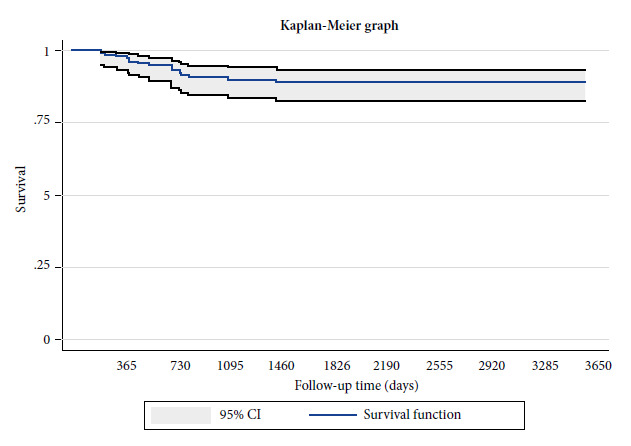
Survival of patients with differentiated thyroid cancer.

At 1-year follow-up, the survival rate was 98% (95%CI: 93.8-99.3), at 2 years it was 93% (95%CI: 87.4-96.2), at 3 years it was 90.1% (95%CI: 83.9-94), at 4 years it was 89.3% (95%CI: 82.8-93.4) and at 5 years it was 89.3% (95%CI 82.8-93.4) (Figure 1).

Survival was significantly lower in those with age at diagnosis ≥ 55 years, tumor size > 4cm, extra-thyroidal extension, distant metastasis, and was higher in those with radioactive iodine treatment (Table 2).

## DISCUSSION

In our study, differentiated thyroid cancer was more frequent in women with papillary histological type and more than half of the patients required total thyroidectomy and the use of radioactive iodine. Survival at 5 years was 89%, being lower in those with TNM stage IV.

Our study analyzed differentiated thyroid cancer, being papillary thyroid cancer, the most frequent type, in agreement with reports from other countries ^5^^,^^6^^,^^12^. This type of cancer affected women more frequently, as in other studies ^5^^,^^6^^,^^12^. This difference between both sexes can possibly be explained by a greater use of health services by women ^13^, and therefore a greater detection of this cancer in asymptomatic stages when the tumors are small. The hormonal effect of estrogen on thyroid stimulating hormone has also been considered, especially in older women with menopause ^14^; however, this is not yet conclusive ^15^.

The mean age at diagnosis (48.3 years) was similar to that reported in Ecuador (44.6 years) ^16^, Brazil (46.5 years) ^5^, Spain (48.3 years) ^6^ and lower than that reported in Colombia (51.1 years) ^12^. These differences may be explained by the cancer detection strategies that have been implemented in each country, such as the use of low-cost imaging techniques that are accessible to the population such as neck ultrasound ^6^^,^^16^, producing a diagnosis at earlier ages and even when the patient has no symptoms ^2^. We found that 30.7% had no alterations on physical examination and detection was incidental, less than in a study in Ecuador, in which 54.2% had this characteristic ^16^. Symptomatic patients at diagnosis tend to present advanced stages of the disease ^17^, while asymptomatic patients are those who have thyroid tumor as a finding incidentally during routine studies, which also explains the increase in diagnosis in recent years ^2^^,^^18^.

In 2016, the TNM staging system for thyroid cancer was updated and one of the important changes was the age cut-off point from 45 to 55 years due to no difference in survival at 10-year follow-up ^11^. In our study, those with age ≥ 55 years had lower survival, similar to other reports ^5^^,^^6^. The TNM stage I (74.2%) in our study was similar to that reported in Brazil (74.8%) ^5^ and lower than that reported in Colombia (82.6%) ^12^, suggesting that the diagnosis of patients is becoming increasingly frequent in early stages, possibly due to the use of imaging resources, such as ultrasound, as a consequence of greater access to medical care ^2^.

The survival rate at five years was 89.3%, lower than that reported in Brazil, 95.8% ^5^, and in Spain 95.1%, at 5 and 15 years of follow-up respectively ^6^. These differences can be explained by conditions that may predispose patients to mortality, since in Brazil and Spain, distant metastasis was reported in 5.3% and 5.5%, lower than the 18% reported in our study. Distant metastasis is found in advanced stages of cancer, being a factor associated with mortality ^5^^,^^6^, and the study showed that these patients had lower survival rates, since involvement of other organs such as the lung or bone may predispose to complications during follow-up. Differences between the health systems of each country ^5^^,^^6^ and the socioeconomic level could also influence survival due to this cancer ^19^.

In our study, the patients who used radioactive iodine had greater survival, contrasting with a previous study in Brazil ^5^. This therapy has two functions, the ablation of benign thyroid remnants after total thyroidectomy, in order to properly interpret serum thyroglobulin (useful in follow-up) and as an adjuvant treatment to eliminate cancerous tissue remnants, thus reducing the risk of recurrence and improving survival ^20^, therefore radioiodine is recommended for patients with regional lymph node metastasis, vascular invasion, extra-thyroidal extension and distant metastasis ^9^.

The prevention, diagnosis and management of cancer is multidisciplinary and requires the participation of all social and governmental actors. In Peru, strategies have been developed to support cancer care, such as the creation of Regional Institutes for Neoplastic Diseases, oncology services and units in hospitals ^21^, implementation and funding of the “Plan Esperanza”, the latter with the aim of reducing the gap in access to oncology services in all regions ^22^, and therefore, mortality from this and other types of cancer.

One of the strengths of this study is that the median follow-up (4.6 years) was similar to studies with a larger number of participants ^5^^,^^6^, which shows the results of the management and follow-up of these patients in the long term. There are also limitations, such as the small sample size, the retrospective collection of data and the fact that we used information from a single hospital, so these results could not be extrapolated to other populations. However, these findings are important because they come from a population outside the capital of Peru, so this topic should be further researched in our country with larger populations, multicenter type, with other characteristics that may influence survival and with more complex designs.

In conclusion, in a Peruvian hospital, differentiated thyroid cancer was more frequent in women in its papillary form, and survival was lower than described in other studies. We recommend prospective studies to be carried out, with longer follow-up time in other Peruvian institutions, in order to compare the clinical outcomes of these patients over time.
