# Supplementary material for: Characteristics and survival of adults with differentiated thyroid cancer in a Peruvian hospital
Source: Rev Peru Med Exp Salud Publica. 2024 Aug 28;41(3):287–93. doi: 10.17843/rpmesp.2024.413.13378 (PMC11495931; doi:10.17843/rpmesp.2024.413.13378)
Supplement: Translation

# Translation: Características y supervivencia de adultos con cáncer diferenciado de tiroides en un hospital de Perú

**Authors:** Luz Morales-Concha, Iván Huamani-Linares, Katy Saihua-Palomino, Edward Luque Florez, Alexi Chávez Echevarría, Ramiro Jorge Tupayachi Palomino, Carlos Antonio Zea Nuñez, Christian R. Mejia, Noé Atamari-Anahui

**Affiliations:** 1 Facultad de Medicina Humana, Universidad Nacional de San Antonio Abad del Cusco, Cusco, Perú.; 2 ASOCIEMH-CUSCO, Facultad de Medicina Humana, Universidad Nacional de San Antonio Abad del Cusco, Cusco, Perú.; 3 Departamento de Cirugía General, Hospital Antonio Lorena, Cusco, Perú.; 4 Hospital Nacional Adolfo Guevara Velasco - EsSalud, Cusco, Perú.; 5 Universidad Continental, Huancayo, Perú.; 6 Asociación Médica de Investigación y Servicios en Salud, Lima, Perú.; 7 Unidad de Investigación para la Generación y Síntesis de Evidencias en Salud, Vicerrectorado de Investigación, Universidad San Ignacio de Loyola, Lima, Perú.

**Keywords:** Cáncer de Tiroides, Tasa de Supervivencia, Mortalidad, Perú

## Abstract

El propósito del estudio fue describir las características clínicas, anatomopatológicas, tratamiento y supervivencia de los pacientes con cáncer de tiroides diferenciado. Se realizó un estudio de cohorte retrospectiva con datos de 150 pacientes de un hospital peruano entre los años 2010 al 2020. Se describieron las características y supervivencia (método de Kaplan-Meier). La media de edad fue 48,3 años, 130 (86,7%) fueron mujeres y el tipo histológico más frecuente fue el papilar 94,6%. El 74,2% tenían estadio TNM I, 70,7% tiroidectomía total y 68,7% recibió yodo radiactivo. La supervivencia global a los 5 años fue 89,3%, siendo menor en aquellos con estadio TNM IV y mayor en los que usaron yodo radiactivo. En conclusión, en un hospital de Cusco, el cáncer diferenciado de tiroides fue más frecuente en mujeres y la supervivencia fue menor en comparación con reportes de otros países.

## INTRODUCCIÓN

El cáncer de tiroides es la neoplasia más frecuente del sistema endocrino ^1^. Según el tipo histológico se puede dividir en tipo diferenciado, medular y anaplásico ^1^. El cáncer de tiroides de tipo diferenciado representa el 90% de todos los casos e incluye al tipo papilar y folicular ^2^.

Según el estudio GLOBOCAN, que recopiló registros de cáncer de tiroides de 185 países, para el año 2020, la incidencia global fue mayor en mujeres que en varones (10,1 casos vs. 3,1 casos por 100 000 habitantes-año) ^3^; al igual que la mortalidad (0,5 muertes por 100 000 habitantes en mujeres y 0,3 muertes por 100 000 habitantes en varones) ^3^. En Perú, para el 2022 se estimó una incidencia de 6,3 casos por 100 000 habitantes-año, ocupando el décimo lugar de todos los cánceres en general y una mortalidad de 0,9 muertes por 100 000 habitantes ^4^.

 Debido al incremento de la incidencia del cáncer de tiroides en todo el mundo, posiblemente por la detección de tumores pequeños y de bajo riesgo como consecuencia de una mayor vigilancia, pero también por la presencia de algunos factores de riesgo, como la exposición a la radiación ionizante ambiental o terapéutica, antecedentes familiares y la obesidad ^2^. El tratamiento en adultos es precoz y oportuno obteniéndose una mayor sobrevida a mediano y largo plazo. La supervivencia del cáncer diferenciado de tiroides es mayor al 95% a los cinco años ^5^, y hasta los 15 años ^6^; sin embargo, en el seguimiento pueden presentar persistencia o recurrencia del cáncer, lo cual genera una vigilancia intensiva y otras opciones del tratamiento ^7^.

En Perú, existen estudios de este tipo de cáncer que se limitan a la descripción de esta neoplasia en el diagnóstico ^8^^-^^10^, dejando un vacío en la evolución y sobrevida de los pacientes, el cual tendría relevancia especialmente en aquellos con recurrencia de la enfermedad, estadio avanzado o metástasis. Por la creciente incidencia de esta neoplasia, decidimos realizar este estudio con el objetivo de describir las características de los pacientes con cáncer diferenciado de tiroides y la supervivencia en un hospital de Cusco.

**MENSAJES CLAVE**

Motivación para realizar el estudio. Existen pocos estudios clínicos y de supervivencia en Perú sobre el cáncer de tiroides.

Principales hallazgos. Entre los años 2010 al 2020, el cáncer diferenciado de tiroides fue más frecuente en mujeres con un estadio precoz de enfermedad, pero la supervivencia fue menor a los cinco años comparado con reportes de otros países.

Implicancias. El cáncer de tiroides se ha incrementado en las últimas décadas en todo el mundo. Es importante disponer de centros especializados y descentralizados para el manejo inicial y seguimiento de estos pacientes para evitar complicaciones o desenlaces fatales a largo plazo y disponer de información epidemiológica actualizada.

## EL ESTUDIO

### Diseño y población

Se realizó un estudio de cohorte retrospectivo, con datos de las historias clínicas de los pacientes con cáncer de tiroides diagnosticados por primera vez en el Hospital Nacional Adolfo Guevara Velasco de Cusco (perteneciente a la seguridad social de Perú); entre el 1 de enero del 2010 al 31 de diciembre del 2016, con tratamiento y seguimiento hasta el 31 de diciembre del 2020.

Se incluyeron todas las historias clínicas de pacientes mayores de 18 años, con confirmación histológica de cáncer diferenciado de tiroides (papilar y folicular) en el servicio de patología del hospital. De 170 pacientes en el periodo de estudio, se excluyeron 20 pacientes: 17 pacientes manejados inicialmente en otras instituciones y tres con diferente tipo histológico (2 medular y 1 anaplásico). El muestreo fue consecutivo de todos los pacientes elegibles siendo la muestra final de 150 historias clínicas. 

### Variables de estudio

Las variables estudiadas fueron sociodemográficas, como el sexo (femenino y masculino), la edad al diagnóstico (20-54 y ≥ 55 años), manifestaciones clínicas (dolor en cuello, disfagia, disfonía, disnea y otros síntomas no relacionados al cáncer de tiroides), características del examen físico de la tiroides (sin alteraciones, nódulo tiroideo y bocio multinodular), función tiroidea inicial (eutiroideo, hipotiroideo e hipertiroideo) a través de la medición de la hormona estimulante de la tiroides, tiroxina libre y triyodotironina.

También se evaluaron características anatomopatológicas como tamaño del tumor, tipo histológico (papilar o folicular), extensión extratiroidea (sí y no), metástasis a ganglio linfático regional (sí y no), metástasis a distancia (sí y no) y estadio TNM (tumor, ganglios linfáticos y metástasis) octava edición (I, II, IV) ^11^, características del tratamiento como tipo de cirugía (tiroidectomía total o lobectomía), linfadenectomía cervical (sí y no), uso de yodo radiactivo (sí y no), características posquirúrgicas (hipotiroidismo, hipoparatiroidismo y lesión del nervio laríngeo recurrente), características del seguimiento (persistencia, recurrencia o excelente respuesta al tratamiento), la supervivencia, que se definió como la duración (días) desde la fecha de diagnóstico hasta el fallecimiento asociado al cáncer y estado final (fallecido o vivo).

### Procedimientos y análisis estadístico

Una vez identificadas a las 150 historias clínicas incluidas en el estudio se procedió a la revisión y recopilación de la información en el área de archivo de hospital, esto a través de una ficha de recolección de datos, previa aprobación y autorización del proyecto por el comité de investigación institucional del hospital. Luego se procedió a depositar la información en una base de datos elaborada en el programa Microsoft Excel ® para Windows 10. Una vez obtenida la base de datos, fue revisada por dos investigadores, para luego extraer y realizar el procesamiento de la información en el programa Stata v.17 (StataCorp LP, College Station, TX, USA).

Las variables categóricas se describieron mediante frecuencias (absolutas y relativas), y las variables numéricas mediante medidas de tendencia central (media o mediana) y de dispersión (rango y desviación estándar); esto de acuerdo con la evaluación previa de la normalidad de los datos mediante la prueba estadística de Shapiro-Wilk. Se realizó comparación según el sexo y entre los dos tipos histológicos (papilar y folicular) a través de la prueba de Chi cuadrado o exacta de Fisher para las variables categóricas y T de Student o U de Mann-Whitney para las variables numéricas, se consideró significativas aquellas con valor p<0,05. Se censuró el tiempo de supervivencia de los participantes vivos al final del estudio (31 de diciembre del 2020) y los fallecidos por otras causas (2 participantes). La supervivencia fue estimada por el método de Kaplan-Meier y para evaluar sus diferencias entre los grupos se utilizó la prueba Log-rank, considerando un valor p<0,05. 

### Aspectos éticos

El proyecto se aprobó por el comité de ética del Hospital Nacional Adolfo Guevara Velasco de Cusco (resolución N°74 -GRACU-ESSALUD-2020). Se utilizó una lista anónima antes de realizar el análisis para no identificar a los participantes.

## HALLAZGOS

De los 150 pacientes incluidos en el estudio, 130 (86,7%) fueron mujeres y la relación mujer/varón fue 6,5. La media de edad al diagnóstico fue 48,3 (DE: 12,7 años). En las características clínicas, 39 (26%) tenían dolor en cuello, 23 (15,3%) disfagia, 19 (12,7%) disfonía, 12 (8%) disnea y 83 (55,3%) otros síntomas no relacionados al cáncer de tiroides. Al examen físico, 46 (30,7%) tenían tiroides sin alteraciones, 59 (39,3%) nódulo tiroideo y 45 (30,0%) bocio multinodular. Con respecto a la función tiroidea, 92 (61,3%) eran eutiroideos, 53 (35,3%) tenían hipotiroidismo y cinco (3,3%) hipertiroidismo. La mediana del tamaño del tumor primario fue 2,5 cm (rango intercuartílico 1,5 a 3,7), 51 (34%) tuvieron extensión extratiroidea, 86 (57,3%) metástasis a ganglio linfático regional, 23/128 (18%) metástasis a distancia y 95 (74,2%) tenían estadio TNM I (octava edición).

 La edad al diagnóstico fue mayor en varones que en mujeres (56,5 años vs. 47 años, p=0,002). Según el tipo histológico, el papilar fue el más frecuente, 142 (94,7%) pacientes y el folicular en 8 (5,3%) pacientes. No se encontraron diferencias significativas entre el tamaño de tumor, extensión extratiroidea, metástasis a ganglio linfático regional, metástasis a distancia y el estadio TNM (Tabla 1).

**Tabla 1:** Características clínicas de los pacientes con cáncer diferenciado de tiroides según tipo histológico y sexo.

Características		Papilar	Folicular	Valor de p ^c^	Masculino	Femenino	Valor de p ^c^
		n (%)	n (%)		n (%)	n (%)	
Sexo				0,598			
	Femenino	122 (85,9)	8 (100,0)		--	--	
	Masculino	20 (14,1)	0 (0,0)		--	--	
Edad al diagnóstico ^a^		48,4 (12,9)	47,1 (9,3)	0,792 ^d^	56,5 (11,9)	47 (12,4)	0,002 ^d^
	20-54	96 (67,6)	7 (87,5)	0,436	9 (45,0)	94 (72,3)	0,014
	≥ 55	46 (32,4)	1 (12,5)		11 (55,0)	36 (27,7)	
Tamaño del tumor ^b^		25 (15-35)	24 (19-41,5)	0,579 ^e^	25,5 (20-42,5)	25 (15-35)	0,218 ^e^
	≤ 4,0 cm	120 (84,5)	6 (75,0)	0,615	15 (75,0)	111 (85,4)	0,321
	> 4,0 cm	22 (15,5)	2 (25,0)		5 (25,0)	19 (14,6)	
Extensión extra tiroidea				0,717			0,265 ^f^
	No	93 (65,5)	6 (75,0)		11 (55,0)	88 (67,7)	
	Sí	49 (34,5)	2 (25,0)		9 (45,0)	42 (32,3)	
Metástasis a ganglio linfático regional				1,000			0,456
	No	61 (43,0)	3 (37,5)		7 (35,0)	57 (43,9)	
	Sí	81 (57,0)	5 (62,5)		13 (65,0)	73 (56,1)	
Metástasis a distancia (n=128)				0,154			0,471
	No	100 (83,3)	5 (62,5)		11 (73,3)	94 (83,2)	
	Sí	20 (16,7)	3 (37,5)		4 (26,7)	19 (16,8)	
Estadio TNM (n=128)				0,260			0,056
	I	90 (75,0)	5 (62,5)		9 (60,0)	86 (76,1)	
	II	17 (14,2)	3 (37,5)		2 (13,3)	18 (15,9)	
	IVA	1 (0,8)	0 (0,0)		1 (6,7)	0 (0,0)	
	IVB	12 (10,0)	0 (0,0)		3 (20,0)	9 (8,0)	

a Media (desviación estándar), ^b^ mediana (rangos intercuartílicos), ^c^ prueba exacta de Fisher, ^d^ prueba T-Student, ^e^ prueba U Mann-Whitney, ^f^ prueba Chi cuadrado.

Con respecto al tratamiento, 106 (70,7%) tuvieron tiroidectomía total, 63 (42%) tuvieron disección del cuello con linfadenectomía (31 disección radical modificada, 22 disección selectiva -20 disección lateral y 2 posterolateral- y 10 disección central). Posterior a la cirugía, 146 (97,3%) tuvieron hipotiroidismo, 58 (38,7%) hipoparatiroidismo y 23 (15,3%) lesión del nervio laríngeo recurrente. Ciento tres (68,7%) pacientes recibieron yodo radiactivo.

La mediana del tiempo de seguimiento fue 4,6 años (RIC 3,5-6,6), siendo para el grupo que terminó el seguimiento (vivos) de 4,9 años (RIC: 3,9-6,8) y aquellos que presentaron el desenlace (fallecidos) de 1,9 años (RIC: 1-2).

Al finalizar el seguimiento se reportaron 15 fallecidos por cáncer (13 papilar y 2 folicular). De estos, 11 (73,3%) fueron ≥ 55 años, todos tuvieron metástasis a ganglio linfático regional y 12 (85,7%) metástasis a distancia (Tabla 2). De los 13 pacientes fallecidos con cáncer papilar, todos tuvieron metástasis a ganglio regional y 10 metástasis a distancia, y los dos pacientes fallecidos con cáncer folicular presentaron también estas características. De los 133 pacientes que no presentaron el desenlace (vivos), 44 (33,1%) tenían persistencia del cáncer, 21 (15,8%) recurrencia y 68 (51,1%) excelente respuesta al tratamiento.

**Tabla 2:** Supervivencia de los pacientes con cáncer diferenciado de tiroides.

Características		Casos	Fallecidos por cáncer	Probabilidad de supervivencia	Log-rank
		n (%)	n (%)	S(t) IC95%	
Sexo					
	Femenino	130 (86,7)	11 (73,3)	90,9 (84,2-94,9)	0,121
	Masculino	20 (13,3)	4 (26,7)	79,0 (53,2-91,5)	
Edad al diagnóstico					
	20-54	103 (68,7)	4 (26,7)	95,8 (89,0-98,4)	<0,001
	≥ 55	47 (31,3)	11 (73,3)	75,0 (59,4-85,3)	
Tipo histológico					
	Papilar	142 (94,7)	13 (86,7)	87 (76,6-93,0)	0,175
	Folicular	8 (5,3)	2 (13,3)	56,3 (14,7-84,2)	
Tamaño del tumor					
	≤ 4,0 cm	126 (84,0)	7 (46,7)	94,0 (87,8-97,1)	0,001
	> 4,0 cm	24 (16,0)	8 (53,3)	63,1 (39,3-79,7)	
Extensión extratiroidea					
	No	99 (66,0)	4 (26,7)	95,4 (88,2-98,3)	<0,001
	Sí	51 (34,0)	11 (73,3)	77,4 (62,9-86,8)	
Metástasis a ganglio linfático regional					
	No	64 (42,7)	0 (0,0)	100 (100,0)	<0,001
	Sí	86 (57,3)	15 (100,0)	81,6 (71,2-88,5)	
Metástasis a distancia (n=128)					
	No	105 (82,0)	2 (14,3)	98,0 (92,3-99,5)	<0,001
	Sí	23 (18,0)	12 (85,7)	45,8 (24,7-64,6)	
Estadio TNM (n=128)					
	I	95 (74,2)	1 (7,2)	99,0 (92,8-99,9)	<0,001
	II	20 (15,6)	3 (21,4)	81,7 (53,1-93,7)	
	IV	13 (10,2)	10 (71,4)	23,1 (5,6-47,5)	
Cirugía					
	Tiroidectomía total	106 (70,7)	12 (80,0)	87,9 (79,6-92,9)	0,42
	Lobectomía	44 (29,3)	3 (20,0)	92,6 (78,7-97,5)	
Linfadenectomía					
	No	87 (58,0)	4 (26,7)	95,1 (87,5-98,1)	0,01
	Sí	63 (42,0)	11 (73,3)	81,3 (68,7-89,2)	
Yodo radiactivo					
	No	47 (31,3)	11 (73,3)	74,6 (58,6-85,1)	<0,001
	Sí	103 (68,7)	4 (26,7)	95,9 (89,4-98,4)	

TNM: tumor, node y metastasis.

Al año de seguimiento, hubo un 98% de supervivencia (IC95%: 93,8-99,3), a los dos años el 93% (IC95%: 87,4-96,2), a los 3 años 90,1% (IC95%: 83,9-94), a los 4 años 89,3% (IC95%: 82,8-93,4) y a los 5 años 89,3% (IC95% 82,8-93,4) (Figura 1).

**Figura 1:** Supervivencia de pacientes con cáncer diferenciado de tiroides.

La supervivencia fue significativamente menor en aquellos con edad al diagnóstico ≥ 55 años, tamaño de tumor > 4cm, extensión extra tiroidea, metástasis a distancia, y fue mayor en aquellos con tratamiento de yodo radiactivo (Tabla 2).

## DISCUSIÓN

En nuestro estudio, el cáncer diferenciado de tiroides fue más frecuente en mujeres con tipo histológico papilar y más de la mitad de los pacientes requirieron tiroidectomía total y uso de yodo radiactivo. La supervivencia a los 5 años fue del 89%, siendo menor en aquellos con estadio TNM IV.

En el estudio se analizó el cáncer de tiroides de tipo diferenciado, siendo el cáncer de tiroides papilar el más frecuente encontrado, concordante con reportes de otros países ^5^^,^^6^^,^^12^. Este tipo de cáncer afectó con mayor frecuencia a las mujeres, al igual que otros estudios ^(^^5^^,^^6^^,^^12^. Esta diferencia entre ambos sexos puede explicarse posiblemente por un mayor uso de los servicios de salud por parte de las mujeres ^13^, y por consiguiente una mayor detección de este cáncer en etapas asintomáticas cuando los tumores son pequeños. También se ha postulado el efecto hormonal del estrógeno sobre la hormona estimulante de la tiroides, especialmente en mujeres con edad más avanzada de la menopausia ^14^; sin embargo, esto aun no es concluyente ^15^.

La media de edad al diagnóstico (48,3 años) fue similar a lo reportado en Ecuador (44,6 años) ^16^, Brasil (46,5 años) ^5^, España (48,3 años) ^6^^)^ y menor a lo reportado en Colombia (51,1 años) ^12^. Posiblemente estas diferencias son explicadas por las estrategias de detección del cáncer que se han ido implementando en cada país, como la realización de técnicas de imagen de bajo costo y accesibles a la población como la ecografía del cuello ^6^^,^^16^, produciendo un diagnóstico a edades más tempranas e, incluso, cuando el paciente no tiene síntomas ^(^^2^. El 30,7% no tuvieron alteraciones al examen físico y la detección fue incidental, menor a un estudio en Ecuador, donde el 54,2% tuvo esta característica ^16^. Los pacientes sintomáticos al diagnóstico tienden a presentar estadios avanzados de la enfermedad ^17^, en cambio los pacientes asintomáticos son los que incidentalmente en estudios de rutina tienen al tumor tiroideo como un hallazgo esto también explica su incremento del diagnóstico en los últimos años ^2^^,^^18^.

El año 2016, se actualizó el sistema de estadificación TNM del cáncer de tiroides y uno de los cambios importantes fue el punto de corte de la edad de 45 a 55 años debido a que no hay diferencias en la supervivencia a los 10 años de seguimiento ^11^. En el estudio, aquellos con edad ≥ 55 años tuvieron menor sobrevida, similar a otros reportes ^5^^,^^6^. El estadio TNM I (74,2%) en el estudio fue similar a lo reportado en Brasil (74,8%) ^5^ y menor a lo reportado el Colombia (82,6%) ^12^, lo que sugiere que el diagnóstico de los pacientes cada vez está siendo frecuente en etapas tempranas posiblemente por el uso de recursos imagenológicos, como la ecografía, consecuencia de un mayor acceso a la atención médica ^(^^2^.

La supervivencia a los cinco años fue 89,3%, menor a lo reportado en Brasil, 95,8% ^5^, y en España 95,1%, a los 5 y 15 años de seguimiento respectivamente ^6^. Estas diferencias se pueden explicar por las condiciones que pueden predisponer a mortalidad en los pacientes, pues en Brasil y España, la metástasis a distancia se reportó en 5,3% y 5,5%, inferior a lo reportado en nuestro estudio 18%. La metástasis a distancia se observa en estadios avanzando del cáncer, siendo un factor asociado a mortalidad ^5^^,^^6^, y en el estudio se evidenció que estos pacientes tenían menor sobrevida, pues el compromiso a otros órganos como el pulmón o el hueso pueden predisponer a complicaciones en el seguimiento. También las diferencias entre los sistemas de salud de cada país ^5^^,^^6^ y el nivel socioeconómico podrían influir en la sobrevida por este cáncer ^19^.

En nuestro estudio, los pacientes que usaron yodo radiactivo tuvieron mayor sobrevida, esto contrastando con un estudio previo en Brasil ^5^. Esta terapia tiene dos funciones, la ablación de restos tiroideos benignos después de una tiroidectomía total, esto para una adecuada interpretación de la tiroglobulina sérica (útil en el seguimiento) y como tratamiento adyuvante para eliminar los restos de tejido canceroso, para así disminuir el riesgo de recurrencia y mejorar la supervivencia ^20^, por eso el yodo radiactivo está recomendado para pacientes con metástasis a ganglios regionales, invasión vascular, extensión extra tiroidea y metástasis a distancia ^9^.

La prevención, diagnóstico y manejo del cáncer es multidisciplinario y requiere la participación de todos los actores sociales y gubernamentales. En Perú, se han desarrollado estrategias que ayudan a la atención oncológica, como la creación de Institutos Regionales de Enfermedades Neoplásicas, servicios y unidades en oncología en los hospitales ^21^, implementación y financiamiento del “Plan Esperanza”, este último con la finalidad de disminuir la brecha de acceso a los servicios oncológicos en todas las regiones ^(^^22^, y por ende, la mortalidad de este y otros tipos de cáncer.

Como fortaleza se debe mencionar que la mediana de seguimiento (4,6 años) fue similar con estudios de mayor cantidad de participantes ^5^^,^^6^, lo que evidencian el resultado del manejo y seguimiento de estos pacientes a largo plazo. También se tienen limitaciones, como una muestra no tan grande, la recopilación retrospectiva de los datos y que provienen de una sola institución hospitalaria, por lo que, estos resultados no se podrían extrapolar a otras poblaciones. Sin embargo, estos hallazgos son importantes, debido a que provienen de una población externa a la capital de Perú, por lo que, este tema se debe seguir investigando en nuestro país con poblaciones más grandes, de tipo multicéntricas, con otras características que puedan influir en la sobrevida y con diseños más complejos.

En conclusión, en un hospital peruano el cáncer diferenciado de tiroides fue más frecuente en mujeres y en su presentación de tipo papilar, la supervivencia fue menor a lo descrito en otros estudios. Recomendamos realizar estudios prospectivos, con mayor tiempo de seguimiento en otras instituciones peruanas; para comparar a través del tiempo los desenlaces clínicos de estos pacientes.
